# Editorial: Novel therapeutic approaches to treat neuroinflammation in the central nervous system

**DOI:** 10.3389/fphar.2023.1326774

**Published:** 2023-11-01

**Authors:** Nadezhda A. German

**Affiliations:** Department of Pharmaceutical Sciences, Jerry H. Hodge School of Pharmacy, Texas Tech University Health Sciences Center, Amarillo, TX, United States

**Keywords:** neuroinflammation, microglia, depression, e-cigarets, ketamine, cognitive impairment

We are excited to present *Novel Therapeutic Approaches to Treat Neuroinflammation in the CNS* as a Research Topic for Frontiers in Pharmacology. The topic is focused on neuroinflammation as a marker of multiple pathological conditions. Studies on the mechanisms associated with CNS-centered inflammation provide us with the knowledge to design safer and more effective therapeutics for treating various diseases.

Our issue begins with an original research paper by Hou et al. on the *in vivo* effect of Chinese Dodder seed, a medicinal plant, on the gut-neuroinflammation axis. The study uses a chronic unpredictable stress-induced depression model in mice to assess the effect of plant-derived compounds on multiple markers of inflammation, including proinflammatory cytokines, inflammatory proteins, and microglia activation. The observed anti-inflammatory effects were correlated with the changes in the gut microbiota of treated animals. Finally, Hou et al. stated the importance of the combination effects of chlorogenic acid and hypercin, the main constituents of this plant extract, on the *in vivo* outcomes.

The interplay between metabolic diseases and immune responses, immunometabolic responses, is a significant factor that defines clinical representation and treatment outcomes for multiple disorders, including diabetes and obesity. Further, microglia activation in animals has been linked with neuronal injury and cognitive impairment, possibly explaining higher risks of cognitive dysfunction in patients with diabetes. Zhang et al. report the effect of levetiracetam, an antiepileptic drug, on the cognitive function of diabetic rats. Using the streptozotocin-induced diabetes model, the authors observed dose-dependent enhancement of cognitive function and hippocampus morphology of treated rats, as well as inhibition in microglia activation and CNS inflammation levels. This novel work is the first to report the ability of FDA-approved levetiracetam to prevent microglia polarization and induce mitochondrial M2 transformation as part of the cognitive impairment treatment strategy.

In the comprehensive review by a Norway team of scientists, the potential role of ketamine in the treatment of resistant cases of depression disorders has been assessed from multiple aspects, including neuroinflammation, neurotoxicity, and neurodegeneration. Lullau et al. have shown that multifaceted actions of this polypharmacological CNS agent result in a rapid and potent reduction of depression symptoms, increased stress resilience, and reversal of stress-induced dysfunctions.

Our issue is concluded with the original research article by Archie et al. looking at the bases of postnatal neuroinflammation associated with prenatal e-sig exposure. The data conclusively show high levels of oxidative stress and mitochondrial dysfunction in pups of CD1 mice exposed to the e-cig vapor with 2.4% nicotine. In addition, higher levels of neuronal injury markers were observed in the postnatal brain, linking this publication to the previous work by this research team on the deterioration of motor, learning, and memory function in e-cig-exposed animals. These data warrant extensive studies on the effect of vaping in pregnant women on the levels and longitude of neuronal injury in neonates.

Taken together, the issue of this Research Topic: “*Novel Therapeutic Approaches to Treat Neuroinflammation in the CNS*” provides recent advances in the deconvoluting mechanisms involved in this pathological process and identifying effective therapeutic strategies for the corresponding treatments. We acknowledge the contributions of all authors to make this research volume possible.

